# Effects of Tranexamic Acid on Bleeding in Pediatric Surgeries: A Systematic Review and Meta-Analysis

**DOI:** 10.3389/fsurg.2021.759937

**Published:** 2021-10-13

**Authors:** Yiyong Wei, Yajun Zhang, Tao Jin, Haiying Wang, Jia Li, Donghang Zhang

**Affiliations:** ^1^Department of Anesthesiology, Affiliated Hospital of Zunyi Medical University, Zunyi, China; ^2^Department of Anesthesiology, West China Hospital of Sichuan University, Chengdu, China; ^3^Department of Anesthesiology, Cangzhou Integrated Traditional Chinese and Western Medicine Hospital, Cangzhou, China; ^4^Department of Anesthesiology, Xi'an Jiao Tong University-Affiliated Honghui Hospital, Xi'an, China

**Keywords:** tranexamic acid, pediatric, surgery, blood loss, blood transfusion, meta-analysis

## Abstract

**Background:** Major pediatric surgeries can cause severe intraoperative blood loss. This meta-analysis aims to evaluate the efficacy of tranexamic acid (TXA) in pediatric surgeries.

**Methods:** We searched PubMed, Embase, Web of Science, and Cochrane Library from the conception to March 31, 2021 to identify eligible randomized controlled trials (RCTs) that evaluated the efficacy of TXA in pediatric surgeries. Two reviewers choosed studies, evaluated quality, extracted data, and assessed the risk of bias independently. Mean difference (MD) was calculated as the summary statistic for continuous data. We used a random-effects model to measure mean effects. Data were generated from the corresponding 95% confidence interval (CI) using RevMan 5.3 software. Primary outcomes included intraoperative and postoperative blood loss, red blood cell (RBC) transfusion as well as fresh frozen plasma (FFP) transfusion.

**Results:** Fifteen studies enrolling 1,332 patients were included in this study. The pooled outcomes demonstrated that TXA was associated with a decreased intraoperative (MD = −1.57 mL/kg, 95% CI, −2.54 to −0.60, *P* = 0.002) and postoperative (MD = −7.85 mL/kg, 95% CI, −10.52 to −5.19, *P* < 0.001) blood loss, a decreased intraoperative (MD = −7.08 mL/kg, 95% CI, −8.01 to −6.16, *P* < 0.001) and postoperative (MD = −5.30 mL/kg, 95% CI, −6.89 to −3.70, *P* < 0.001) RBC transfusion, as well as a decreased intraoperative (MD = −2.74 mL/kg, 95% CI, −4.54 to −0.94, *P* = 0.003) and postoperative (MD = −6.09 mL/kg, 95% CI, −8.26 to −3.91, *P* < 0.001) FFP transfusion in pediatric surgeries. However, no significant difference was noted between two groups in duration of surgery (MD = −12.51 min, 95% CI −36.65 to 11.63, *P* = 0.31). Outcomes of intraoperative and postoperative blood loss and the duration of surgery in included studies were not pooled due to the high heterogeneity.

**Conclusion:** This meta-analysis demonstrated that TXA was beneficial for bleeding in pediatric surgeries.

## Introduction

Every year, millions of children are subjected to a variety of surgeries (1). The surgical operation is linked to significant blood loss in the perioperative period, which increases the risk of intraoperative hypotension, anemia, obstructed view of the surgical field, organ (particularly cardiac, renal, and pulmonary) damage, metabolic acidosis, infection, and other morbidities (2). Surgical blood loss and the requisite blood transfusions are a major cause of pediatric surgical mortality due to the complications of tissue hypoperfusion, electrolyte disorders, hemolytic reaction, and infectious diseases (3, 4). Therefore, it is necessary to prevent blood loss and minimize the need for blood transfusion in pediatric surgeries.

The pharmacological approach to reduce bleeding and the consequent need for transfusion have recently become an effective approach to preserve blood in adult patients. Tranexamic acid (TXA), a synthetic derivative of the amino acid lysine, acts by binding at lysine-binding sites and composes a convertible complex with both plasminogen and plasmin. By competitive blocking the transformation of plasminogen to plasmin, the proteolysis of plasmin on fibrin clots and platelets was inhibited, thus inhibiting fibrinolysis at the operative wound (5, 6). Many studies have shown that TXA can effectively reduce intraoperative bleeding in pediatric craniosynostosis, cardiac, scoliosis, adenotonsillectomy, and endoscopic sinus surgery (7–9). However, the results from individual studies of administering TXA in the pediatric population are not completely identical (10). Therefore, we conducted a systematic review and meta-analysis to assess the efficacy of TXA in reducing blood loss and blood transfusion in pediatric surgeries.

## Methods

### Literature Search

This systematic review and meta-analysis was registered in the International Prospective Register of Systematic Reviews (PROSPERO; Registration NO. CRD42020198314) and was conducted according to the Preferred Reporting Items for Systematic Reviews and Meta-Analyses statement issued in 2009 (11). The four electronic databases PubMed, Embase (*via* Ovid), the Cochrane Library, and Web of Science were collected for studies to include in the present meta-analysis. Keywords included “tranexamic acid,” “pediatric,” “child,” “infant,” “toddler,” and “preschool.” The search included all articles published up to March 31, 2021. Any disagreements were resolved by discussion or by consulting the senior authors.

### Inclusion and Exclusion Criteria

We reviewed the titles and abstracts of all retrieved studies. Inclusion criteria: (a) only placebo-controlled randomized controlled trials (RCTs) that described the efficacy of TXA on reducing intraoperative and postoperative bleeding or blood transfusion or duration of surgery in children undergoing surgery were included; (b) studies enrolled only children aged 0–18 years. (c) studies contained sufficient raw data for weighed mean difference (WMD) with 95% confidence intervals (CIs). Reviews, conference abstracts, letters, retrospective or case series, and studies of adult surgery were excluded.

### Data Extraction

The following data were extracted: sample of patients, age, weight, surgery type, TXA dose, blood loss, transfusion of RBCs and FFP intraoperatively and 24 h postoperationly, as well as the duration of surgery.

### Bias and Quality Assessment

Cochrane Collaboration tool (12) was used to assess the quality of included studies. Based on sequence generation, allocation concealment, blinding, data collection, and outcome reporting, we will assign the risk of bias into low risk, high risk, or unclear.

### Data Analysis

The statistical analysis was performed using Review Manager 5.3 software (The Cochrane Collaboration, Oxford, UK). Random-effects models were used to compute the WMD for continuous variables. Heterogeneity was assessed by *I*^2^ statistics, *I*^2^ > 50% and *P* < 0.01 were considered the existence of considerable heterogeneity. In studies with multiple dose groups, each dose group was defined as a separate study and compared with the control. A subgroup analysis was conducted to test the robustness of the pooled results.

## Results

### Study Characteristics

**Figure 1:**
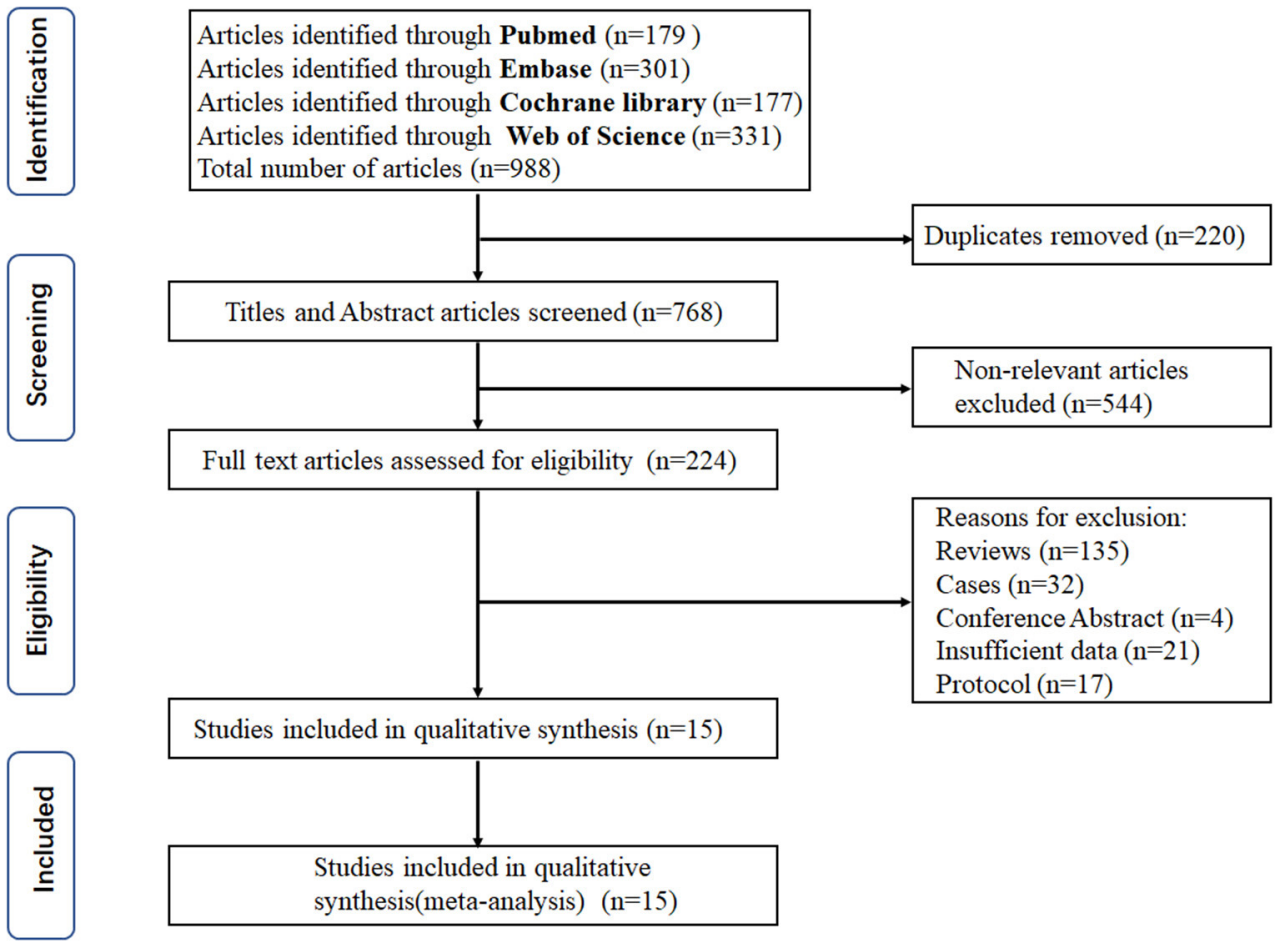
The flow diagram of study selection for this meta-analysis.

The trial selection process is shown in Figure 1. Only 15 studies were eligible according to the inclusion criteria (7–10, 13–23). The characteristics of the included trials are summarized in Table 1. The trials involved cardiac (7 trials) (13–15, 17, 22, 23), craniosynostosis (4 trials) (7, 8, 18, 20), scoliosis (2 trials) (9, 21), adenotonsillectomy (1 trials) (10), and endoscopic sinus (1 trials) (19) surgery. All trials were placebo-controlled. For seven studies (7–9, 18, 20–22), the loading dose of TXA ranged from 10 to 100 mg/kg and maintenance dose ranged from 3 to 15 mg/kg/h; for four studies (10, 14, 19, 23), the loading dose of TXA ranged from 10 to 100 mg/kg and a maintenance dose was not administered; for other four cardiac studies (13, 15–17), TXA was administered thrice at a dosage of 10 mg/kg, before cardiopulmonary bypass (CPB), on CPB, and after CPB, respectively.

**Table 1:** Characteristics of included studies.

**References**	**Age (m)**	**Weight (kg)**	**TXA/control**	**Dose of TXA**	**Surgery type**	**Outcome measures**
Zonis et al. (23)	62.8/52.6	21.3/16.4	40/42	50 mg/kg as a bolus for 1 time	Cardiac surgery	2
Chauhan et al. (16)	52.8/50.4	8.2/7.9	96/24	30 mg/kg as a bolus for 1 time	Cardiac surgery	2, 4, 6
Chauhan et al. (15)	3.3/4.3	6.2/7.0	30/30	50 mg/kg as a bolus for 1 time and then 10 mg/kg as a bolus for 1 time	Cardiac surgery	2, 4, 6
Chauhan et al. (15)	4.2/4.3	5.2/7.0	30/30	10 mg/kg as a bolus followed by 1 mg/kg/h infusion	Cardiac surgery	2, 4, 6
Chauhan et al. (15)	3.0/4.3	6.3/7.0	30/30	10 mg/kg as a bolus for 3 times	Cardiac surgery	2, 4, 6
Chauhan et al. (15)	2.9/4.3	6.6/7.0	30/30	20 mg/kg as a bolus for 2 times	Cardiac surgery	2, 4, 6
Chauhan et al. (17)	49.2/50.4	6.9/7.1	50/50	30 mg/kg as a bolus for 1 time	Cardiac surgery	2, 4, 6
Bulutcu et al. (14)	49.2/45.6	6.2/5.8	25/25	100 mg/kg as a bolus for 1 time	Cardiac surgery	2, 4, 6
Sethna et al. (21)	163/168	59/52	23/21	100 mg/kg as a bolus followed by 10 mg/kg/h infusion	Scoliosis	1, 3, 5, 7
Dadure et al. (18)	7/6	8/8	19/21	15 mg/kg as a bolus followed by 10 mg/kg/h infusion	Craniosynostosis	1, 2, 3, 4, 7
Goobie et al. (20)	23/25	11/11	23/20	50 mg/kg as a bolus followed by 5 mg/kg/h infusion	Craniosynostosis	1, 2, 3, 4, 7
Shimizu et al. (22)	31/31	11/10	81/79	50 mg/kg as a bolus followed by 15 mg/kg/h infusion	Cardiac surgery	2, 3, 5
Aggarwal et al. (13)	38/36	8/9	40/40	30 mg/kg as a bolus for 1 time	Cardiac surgery	2
Brum et al. (10)	77/88	27/32	47/48	10 mg/kg as a bolus for 1 time	Adenotonsillectomy	1
Eldaba et al. (19)	90/86	36/37	50/50	25 mg/kg as a bolus for 1 time	Endoscopic sinsus	1, 7
Kim et al. (8)	12/14	10/11	23/25	10 mg/kg as a bolus followed by 5 mg/kg/h infusion	Craniosynostosis	1, 2, 3, 4, 5, 6, 7
Saleh and Mostafa (9)	175/175	39/39	25/25	50 mg/kg as a bolus followed by 20 mg/kg/h infusion	Scoliosis	1, 7
Saleh and Mostafa (9)	175/175	39/39	25/25	10 mg/kg as a bolus followed by 1 mg/kg/h infusion	Scoliosis	1, 7
Fenger-Eriksen (7)	9/8	9/10	15/15	10 mg/kg as a bolus followed by 3 mg/kg/h infusion	Craniosynostosis	1, 2, 3, 4, 5, 6, 7

*1, intraoperative blood loss; 2, postoperative blood loss; 3, intraoperative RBC; 4, postoperative RBC; 5, intraoperative FFP; 6, postoperative FFP; 7, duration of surgery*.

### Quality of Included Studies

**Figure 2:**
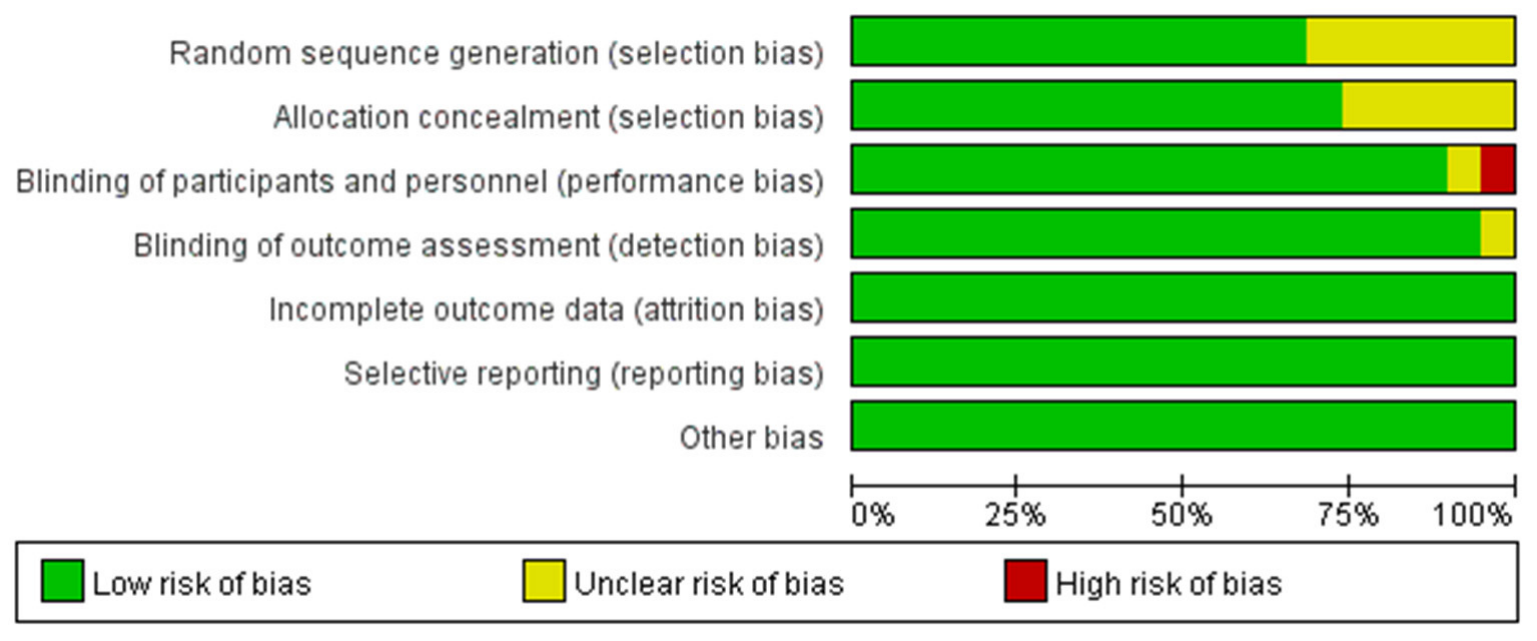
Quality of included studies. The overall quality of the selected studies was assessed by the Cochrane Collaboration tool.

All included studies were double-blind RCTs. Except for three studies (15–17) that were deemed unclear for the method of random allocation, other studies had detailed description of randomization methods using a computer-generated allocation list. Two studies (13, 15) did not mention allocation concealment and were rated as unclear. Information about allocation concealment of two articles (16, 22) was insufficient to allow judgment, and these two articles were rated as unclear (Figure 2).

### Efficacy Outcomes

#### Intraoperative Blood Loss

**Figure 3:**
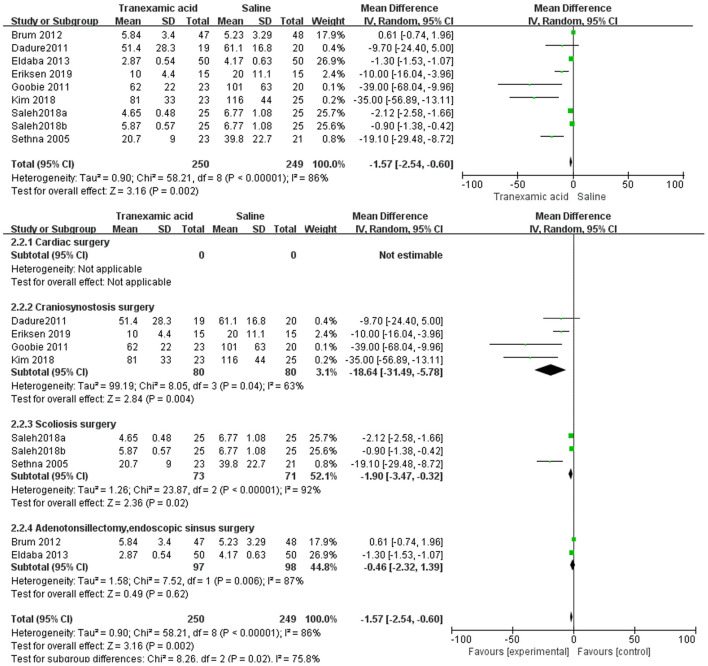
Meta-analysis of intraoperative blood loss for TXA compared with placebo.

Eight studies (7–10, 18–21) had sufficient data to analyze intraoperative blood loss. Using the random-effects model, the pooled MD showed a smaller total intraoperative blood loss in the TXA group vs. the control group (MD = −1.57 mL/kg, 95% CI, −2.54 to −0.60; *I*^2^ = 86%, P-heterogeneity < 0.00001) (Figure 3). The subgroup analyses showed that the effect of TXA on intraoperative blood loss varied by the type of surgery. For craniosynostosis surgery, the MD showed a smaller total intraoperative blood loss in the TXA group vs. the control group (MD = −18.64 mL/kg, 95% CI, −31.49 to −5.78; *I*^2^ = 63%, P-heterogeneity = 0.04) (Figure 3). For scoliosis surgery, the MD showed a smaller total intraoperative blood loss in the TXA group vs. the control group (MD = −1.90 mL/kg, 95% CI, −3.47 to −0.32; *I*^2^ = 92%, P-heterogeneity < 0.00001) (Figure 3). For endoscopic sinus and adenotonsillectomy surgery, there was no difference in intraoperative blood loss in the TXA group vs. the control group (MD = −0.46 mL/kg, 95% CI, −2.32 to 1.39; *I*^2^ = 87%, P-heterogeneity < 0.00001) (Figure 3).

#### Postoperative Blood Loss

**Figure 4:**
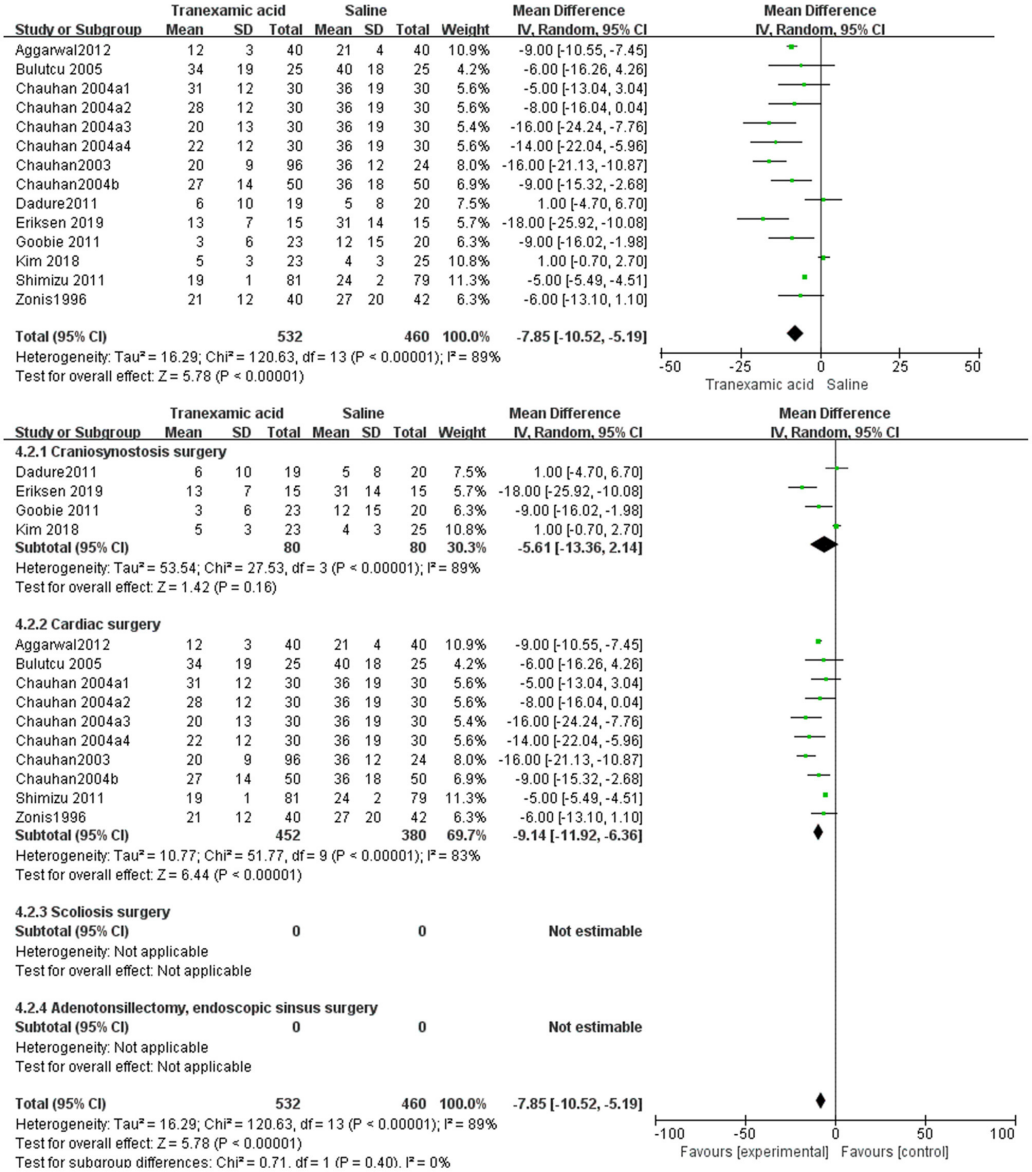
Meta-analysis of blood loss at 24 h after surgery for TXA compared with placebo.

Eleven studies (7, 8, 13–20, 22, 23) had sufficient data to analyze postoperative blood loss. Using the random-effects model, the pooled MD showed a smaller total postoperative blood loss in the TXA group vs. the control group (MD = −7.85 mL/kg, 95% CI, −10.52 to −5.19; *I*^2^ = 89%, P-heterogeneity < 0.00001) (Figure 4). The subgroup analyses showed that the effect of TXA on intraoperative blood loss varied by surgery types. For craniosynostosis surgery, there was no difference in postoperative blood loss in the TXA group vs. the control group (MD = −5.61 mL/kg, 95% CI, −13.36 to 2.14; *I*^2^ = 89%, P-heterogeneity < 0.00001) (Figure 4). For cardiac surgery, the MD showed a smaller total postoperative blood loss in the TXA group vs. the control group (MD = −9.14 mL/kg, 95% CI, −11.92 to −6.36; *I*^2^ = 83%, P-heterogeneity < 0.00001) (Figure 4).

### Intraoperative RBC Transfusion

**Figure 5:**
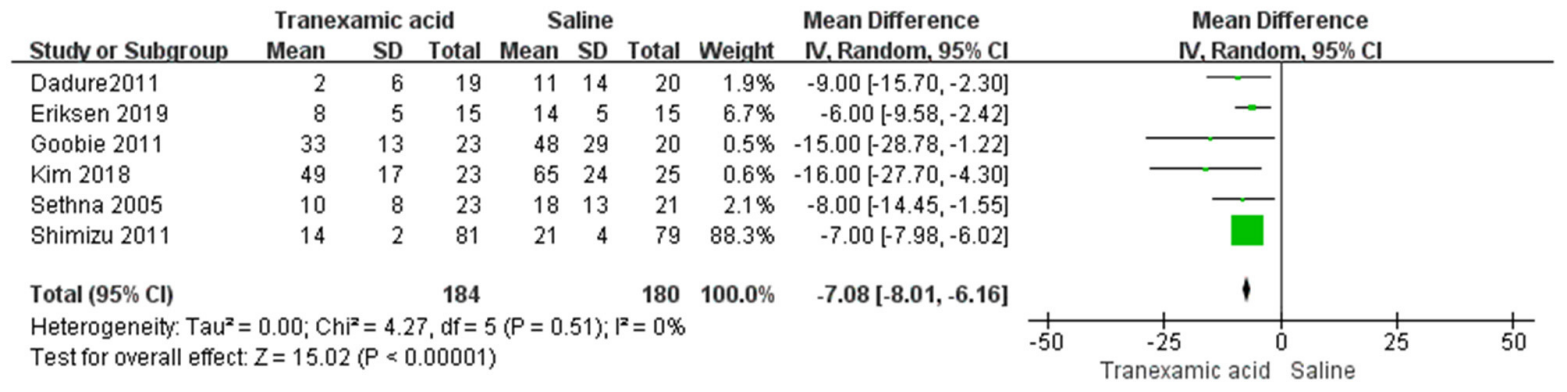
Meta-analysis of intraoperative RBC transfusion for TXA compared with placebo.

Six studies (7, 8, 18, 20–22) had sufficient data to analyze intraoperative RBC transfusion. Using the random-effects model, the pooled MD showed a significantly smaller total intraoperative RBC transfusion in the TXA group vs. the control group (MD = −7.08 mL/kg, 95% CI, −8.01 to −6.16; *I*^2^ = 0%, P-heterogeneity = 0.51) (Figure 5).

### Postoperative RBC Transfusion

**Figure 6:**
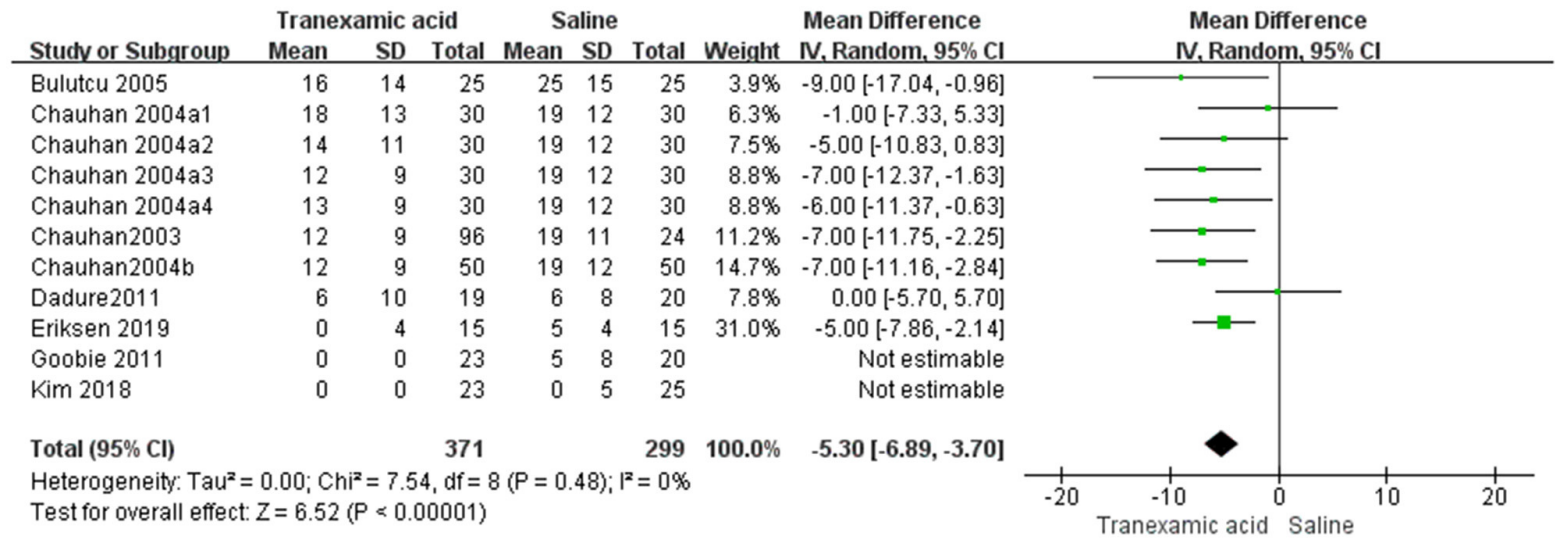
Meta-analysis of RBC transfusion at 24 h after surgery for TXA compared with placebo.

Eight studies (7, 8, 14–18, 20) had sufficient data to analyze postoperative RBC transfusion. Using the random-effects model, the pooled MD showed a smaller total postoperative RBC transfusion in the TXA group vs. the control group (MD = −5.30 mL/kg, 95% CI, −6.89 to −3.70; *I*^2^ = 0%, P-heterogeneity = 0.48) (Figure 6).

### Intraoperative FFP Transfusion

**Figure 7:**
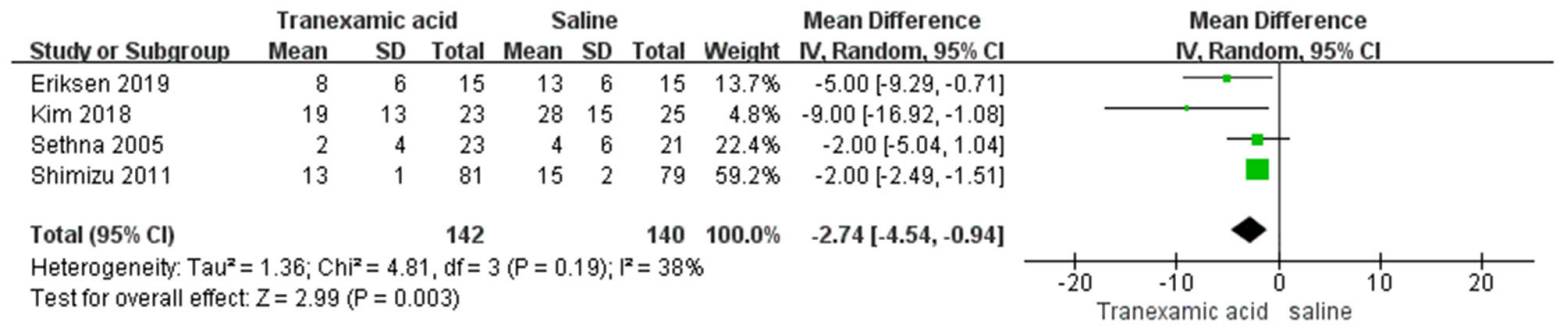
Meta-analysis of intraoperative FFP transfusion for TXA compared with placebo.

Four studies (7, 8, 21, 22) had sufficient data to analyze intraoperative FFP transfusion. Using the random-effects model, the pooled MD showed a smaller total intraoperative FFP in the TXA group vs. the control group (MD = −2.74 mL/kg, 95% CI, −4.54 to −0.94; *I*^2^ = 38%, P-heterogeneity = 0.19) (Figure 7).

### Postoperative FFP Transfusion

**Figure 8:**
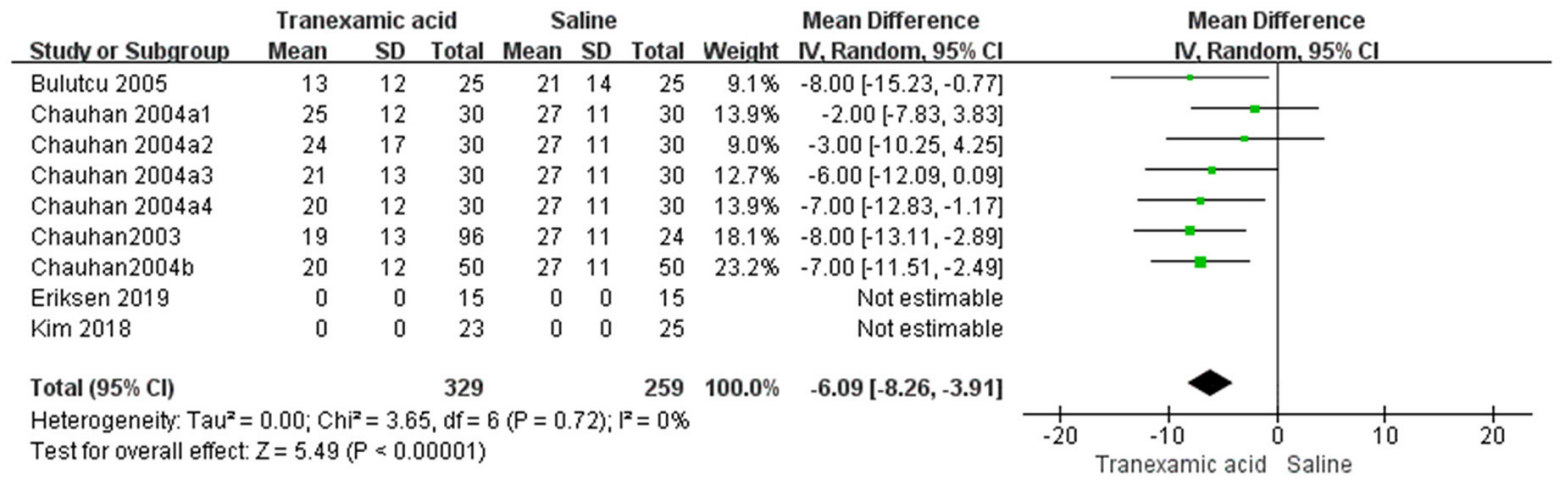
Meta-analysis of FFP transfusion at 24 h after surgery for TXA compared with placebo.

Six studies (7, 8, 14–17) had sufficient data to analyze postoperative FFP transfusion. Using the random-effects model, the pooled MD showed a significantly smaller total postoperative FFP in the TXA group vs. the control group (MD = −6.09 mL/kg, 95% CI, −8.26 to −3.91; *I*^2^ = 0%, P-heterogeneity = 0.72) (Figure 8).

### Duration of Surgery

**Figure 9:**
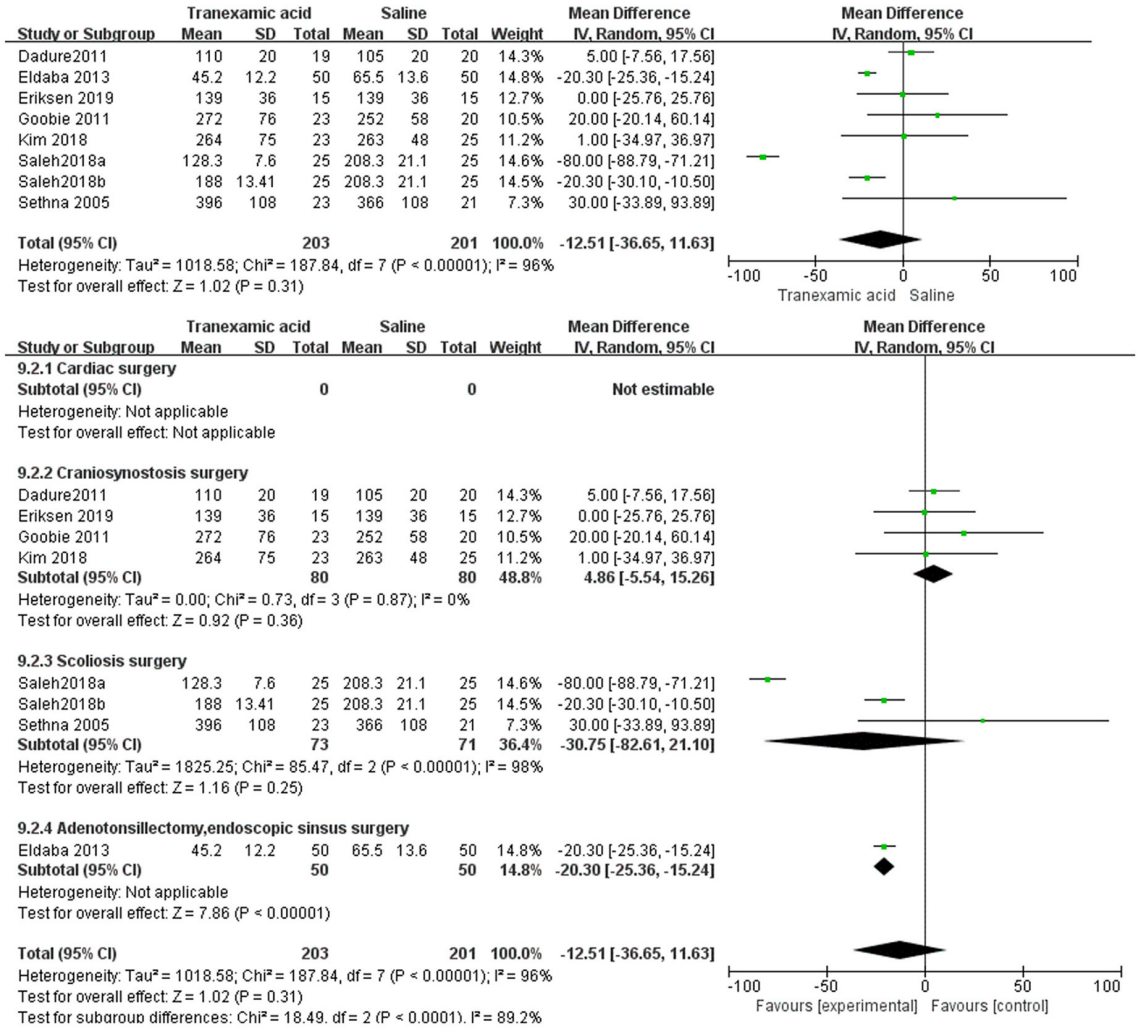
Meta-analysis of duration of surgery for TXA compared with placebo.

Seven studies (7, 9, 18–21) had sufficient data to analyze duration of surgery. The random-effects model found no difference between the TXA and placebo groups (MD = −12.51 min, 95% CI, −36.65 to 11.63; *I*^2^ = 96%, P-heterogeneity < 0.00001) (Figure 9). The subgroup analyses showed that there was no difference in the duration of surgery for the craniosynostosis surgery (MD = 4.86 mL/kg, 95% CI −5.54 to 15.26; *I*^2^ = 0%, P-heterogeneity = 0.87) and scoliosis surgery (MD = −30.75 mL/kg, 95% CI, −82.61 to 21.10; *I*^2^ = 98%, P-heterogeneity < 0.00001) (Figure 9).

## Discussion

Bleeding and blood transfusion were commonly seen in children undergoing cardiac, craniosynostosis and scoliosis surgeries (24–26). The necessary RBC transfusion requirements heightens the risk of transmission of several infectious diseases (27), and seriously burdens hospitals and patients financially. TXA has been studied for its potential to reduce various surgical bleeding. Therefore, it is very meaningful to explore the effects of TXA on the blood loss and the need for blood transfusion in pediatric surgical procedures.

This meta-analysis shows that TXA could reduce intraoperative and postoperative blood loss during all surgery compared with the placebo control. However, after subgroup analysis, we did not find a statistically significant reduction in intraoperative blood loss during pediatric scoliosis, endoscopic sinus, and adenotonsillectomy surgery, nor in postoperative blood loss during pediatric craniosynostosis surgery. Notably, TXA could reduce intraoperative blood loss during pediatric craniosynostosis surgery and reduce postoperative blood loss during pediatric cardiac surgery. The discrepancy is perhaps associated with the small number of included reports and with the differences in the measuring methods of blood loss.

However, it is not appropriate to aggregate these results to assess the effect of TXA in reducing bleeding and blood transfusion because the administered dose of TXA in these studies was different (loading doses from 10 to 100 mg/kg and infusion rates of 1–10 mg/kg per hour) and the experimental approaches were also varied. Moreover, the ages of the children and surgical procedures in these studies were also different.

Our results indicated a decrease of bleeding in the TXA group during surgery. However, there is a non-significant trend toward decrease in bleeding in the treated group compared with the placebo group during pediatric craniosynostosis surgery in postoperative period. Based on our data, it would be interesting to continue use of TXA in children treated for surgical correction of craniosynostosis.

The results of our meta-analysis indicate that TXA decreased postoperative blood loss and RBC and FFP transfusion compared with placebo in pediatric cardiac surgery. However, the outcomes regarding postoperative blood loss were too heterogeneously distributed. Many factors may contribute to the high degree of heterogeneity. The various dosage schemes used in included studies might be the major reasons. After anesthetic induction, some studies performed a single bolus with 10–100 mg/kg (14, 23). Several studies used multiple boluses at different time points: anesthetic induction, CPB priming, and after the administration of protamine (16, 17). While some used continuous infusion of TXA during and/or after CPB (13). One study (22) used 50 mg/kg of TXA as a bolus followed by a 15 mg/kg/h infusion and another 50 mg/kg into the bypass circuit. The TXA dosage used was empirical based on its effects on blood loss instead of based on pharmacodynamic data regarding the fibrinolytic inhibiting activity. Only one study performed by Chauhan and colleagues (15) compared different TXA dosage schemes in a pediatric population. They found that the maximum reduction in blood loss was acquired with a bolus of 10 mg/kg TXA after anesthetic induction and repeated at the initiation and after weaning from CPB, respectively.

The pharmacological data on TXA in the pediatric cardiac surgeries are not available. Pharmacokinetic data in adults indicated a loading dose of 12.5 mg/kg given more than 30 min, a maintenance dose of 6.5 mg/kg/h and a dose of 1 mg/kg for CPB priming is required to maintain TXA concentration higher than 345 μM in blood, which is considered to be the lowest concentration for complete inhibition of fibrinolysis. Through this scheme, the TXA plasma concentration was more stable compared with repeated bolus administration schemes (28). However, these pharmacokinetic data are not directly applicable to a pediatric population.

Adverse effects associated with TXA use were not adequately reported. Our study indicates that TXA reduces postoperative blood loss and blood transfusion need in pediatric cardiac surgery; further follow up studies to assess the effects of TXA on postoperative outcomes and to determine the optimal dosage of TXA are needed.

Two studies (9, 21) indicated that administration of TXA produces significant reduction of blood loss in children with scoliosis. However, there was a clear heterogeneity with *I*^2^ of 92%. One study (21) used 100 mg/kg of TXA as a bolus followed by 10 mg/kg/h infusion. Another (9) used two doses: the high dose was 50 mg/kg of TXA as a bolus followed by 20 mg/kg/h infusion, and the low dose was 10 mg/kg of TXA as a bolus followed by 1 mg/kg/h infusion. Interestingly, all these three doses were found to reduce intraoperative blood loss.

In one study with a dose of 25 mg/kg (19), there was a significant decrease in volume of intraoperative bleeding, and duration of surgery in TXA group as compared to placebo group in pediatric endoscopic sinus surgery. However, in another study with a dose of 10 mg/kg, there was no benefit in the use of TXA for reducing bleeding during the perioperative period of adenotonsillectomy in children (10).

Due to there are two reports in the literature that have described the length of hospital stay (8, 20) and one report in the literature that have described the complications (8), we did not compare with these two indicators in our meta-analysis. After a detailed literature research, no published studies concerning TXA used in the pediatric surgical oncology, pediatric emergency surgery and neonatal surgery, therefore, we did not analyze these surgery type, these issues merit further studies in the near future.

In conclusion, this meta-analysis suggests that TXA contributes to reduce the transfusion of RBC and FFP in children undergoing cardiac, craniosynostosis, scoliosis, and endoscopic sinus surgery. There is, However, controversy over the efficacy of TXA in reducing intraoperative and postoperative blood loss. As a consequence, new RCTs evaluating the effects of TXA in children with these surgeries should be performed.

## Data Availability Statement

The original contributions presented in the study are included in the article/supplementary material, further inquiries can be directed to the corresponding authors.

## Author Contributions

All authors listed have made a substantial, direct and intellectual contribution to the work, and approved it for publication.

## Conflict of Interest

The authors declare that the research was conducted in the absence of any commercial or financial relationships that could be construed as a potential conflict of interest.

## Publisher's Note

All claims expressed in this article are solely those of the authors and do not necessarily represent those of their affiliated organizations, or those of the publisher, the editors and the reviewers. Any product that may be evaluated in this article, or claim that may be made by its manufacturer, is not guaranteed or endorsed by the publisher.

## References

[1] Zhao Z Li B Wu Y Chen X Guo Y Shen Y . Ketamine affects the integration of developmentally generated granule neurons in the adult stage. BMC Neurosci. (2019) 20:60. 10.1186/s12868-019-0542-4 31852437 PMC6921590

[2] Allain J Stramer S Carneiro-Proietti A Martins M Lopes da Silva S Ribeiro M . Transfusion-transmitted infectious diseases. Biologicals. (2009) 37:71–7. 10.1016/j.biologicals.2009.01.002 19231236

[3] Vamvakas E. Long-term survival rate of pediatric patients after blood transfusion. Transfusion. (2008) 48:2478–80. 10.1111/j.1537-2995.2008.01921.x 19054379

[4] Vamvakas E Blajchman M. Transfusion-related mortality: the ongoing risks of allogeneic blood transfusion and the available strategies for their prevention. Blood. (2009) 113:3406–17. 10.1182/blood-2008-10-167643 19188662

[5] McCormack P. Tranexamic acid: a review of its use in the treatment of hyperfibrinolysis. Drugs. (2012) 72:585–617. 10.2165/11209070-000000000-00000 22397329

[6] Wu G Mazzitelli B Quek A Veldman M Conroy P Caradoc-Davies T . Tranexamic acid is an active site inhibitor of urokinase plasminogen activator. Blood Adv. (2019) 3:729–33. 10.1182/bloodadvances.2018025429 30814058 PMC6418500

[7] Fenger-Eriksen C Lindholm AD Norholt SE von Oettingen G Tarpgaard M Krogh L . Reduced perioperative blood loss in children undergoing craniosynostosis surgery using prolonged tranexamic acid infusion: a randomised trial. Br J Anaesth. (2019) 122:760–6. 10.1016/j.bja.2019.02.017 30952386

[8] Kim EJ Kim YO Shim KW Ko BW Lee JW Koo BN. Effects of tranexamic acid based on its population pharmacokinetics in pediatric patients undergoing distraction osteogenesis for craniosynostosis: Rotational thromboelastometry (ROTEM™) analysis. Int J Med Sci. (2018) 15:788–95. 10.7150/ijms.25008 30008588 PMC6036088

[9] Saleh AN Mostafa RH. Increased nociception following administration of different doses of tranexamic acid in adolescent idiopathic scoliosis surgery. Open Anesthesiol J. (2018) 12:61–8. 10.2174/2589645801812010061

[10] Brum MR Miura MS Castro SF Machado GM Lima LH Lubianca Neto JF. Tranexamic acid in adenotonsillectomy in children: a double-blind randomized clinical trial. Int J Pediatr Otorhinolaryngol. (2012) 76:1401–5. 10.1016/j.ijporl.2012.04.028 22704676

[11] Moher D Liberati A Tetzlaff J Altman D. Preferred reporting items for systematic reviews and meta-analyses: the PRISMA statement. PLoS Med. (2009) 6:e1000097. 10.1371/journal.pmed.1000097 19621072 PMC2707599

[12] Higgins J Altman D Gøtzsche P Jüni P Moher D Oxman A . The Cochrane Collaboration's tool for assessing risk of bias in randomised trials. BMJ. (2011) 343:d5928. 10.1136/bmj.d5928 22008217 PMC3196245

[13] Aggarwal V Kapoor PM Choudhury M Kiran U Chowdhury U. Utility of Sonoclot analysis and tranexamic acid in tetralogy of Fallot patients undergoing intracardiac repair. Ann Card Anaesth. (2012) 15:26–31. 10.4103/0971-9784.91477 22234018

[14] Bulutcu FS Ozbek U Polat B Yalcin Y Karaci AR Bayindir O. Which may be effective to reduce blood loss after cardiac operations in cyanotic children: tranexamic acid, aprotinin or a combination? Pediatric Anesthesia. (2005) 15:41–6. 10.1111/j.1460-9592.2004.01366.x 15649162

[15] Chauhan S Bisoi A Kumar N Mittal D Kale S Kiran U . Dose comparison of tranexamic acid in pediatric cardiac surgery. Asian Cardiovasc Thorac Ann. (2004) 12:121–4. 10.1177/021849230401200208 15213077

[16] Chauhan S Bisoi A Modi R Gharde P Rajesh MR. Tranexamic acid in paediatric cardiac surgery. Indian J Med Res. (2003) 118:86–9. 10.1093/fampra/cmg432 14680204

[17] Chauhan S Das SN Bisoi A Kale S Kiran U. Comparison of epsilon aminocaproic acid and tranexamic acid in pediatric cardiac surgery. J Cardiothorac Vasc Anesth. (2004) 18:141–3. 10.1053/j.jvca.2004.01.016 15073700

[18] Dadure C Sauter M Bringuier S Bigorre M Raux O Rochette A . Intraoperative tranexamic acid reduces blood transfusion in children undergoing craniosynostosis surgery a randomized double-blind study. Anesthesiology. (2011) 114:856–61. 10.1097/ALN.0b013e318210f9e3 21358317

[19] Eldaba AA Amr YM Albirmawy OA. Effects of tranexamic acid during endoscopic sinsus surgery in children. Saudi J Anaesth. (2013) 7:229–33. 10.4103/1658-354X.115314 24015121 PMC3757791

[20] Goobie SM Meier PM Pereira LM McGowan FX Prescilla RP Scharp LA . Efficacy of tranexamic acid in pediatric craniosynostosis surgery: a double-blind, placebo-controlled trial. Anesthesiology. (2011) 114:862–71. 10.1097/ALN.0b013e318210fd8f 21364458

[21] Sethna NF Zurakowski D Brustowicz RM Bacsik J Sullivan LJ Shapiro F. Tranexamic acid reduces intraoperative blood loss in pediatric patients undergoing scoliosis surgery. Anesthesiology. (2005) 102:727–32. 10.1097/00000542-200504000-00006 15791100

[22] Shimizu K Toda Y Iwasaki T Takeuchi M Morimatsu H Egi M . Effect of tranexamic acid on blood loss in pediatric cardiac surgery: a randomized trial. J Anesth. (2011) 25:823–30. 10.1007/s00540-011-1235-z 21947753

[23] Zonis Z Seear M Reichert C Sett S Allen C. The effect of preoperative tranexamic acid on blood loss after cardiac operations in children. J Thoracic Cardiovasc Surg. (1996) 111:982–7. 10.1016/S0022-5223(96)70374-4 8622323

[24] Goobie S Zurakowski D Isaac K Taicher B Fernandez P Derderian C . Predictors of perioperative complications in paediatric cranial vault reconstruction surgery: a multicentre observational study from the Pediatric Craniofacial Collaborative Group. Br J Anaesth. (2019) 122:215–23. 10.1016/j.bja.2018.10.061 30686307

[25] Oetgen M Litrenta J. Perioperative blood management in pediatric spine surgery. J Am Acad Orthop Surg. (2017) 25:480–8. 10.5435/JAAOS-D-16-00035 28644187

[26] Siemens K Sangaran D Hunt B Murdoch I Tibby S. Strategies for prevention and management of bleeding following pediatric cardiac surgery on cardiopulmonary bypass: a scoping review. Pediatric Crit Care Med. (2018) 19:40–7. 10.1097/PCC.0000000000001387 29189637

[27] Squires J. Risks of transfusion. South Med J. (2011) 104:762–9. 10.1097/SMJ.0b013e31823213b6 22024787

[28] Dowd N Karski J Cheng D Carroll J Lin Y James R . Pharmacokinetics of tranexamic acid during cardiopulmonary bypass. Anesthesiology. (2002) 97:390–9. 10.1097/00000542-200208000-00016 12151929

